# *Porphyromonas gingivalis* infection promotes inflammation via inhibition of the AhR signalling pathway in periodontitis

**DOI:** 10.1111/cpr.13364

**Published:** 2022-11-29

**Authors:** Ting Hao, Rui Zhang, Tian Zhao, Juan Wu, Wai Keung Leung, Jie Yang, Weibin Sun

**Affiliations:** ^1^ Department of Periodontology, Nanjing Stomatological Hospital Medical School of Nanjing University Nanjing China; ^2^ Faculty of Dentistry The University of Hong Kong Hong Kong SAR China

## Abstract

*Porphyromonas gingivalis* (*P. gingivalis*) is a key pathogen of chronic periodontitis. Aryl hydrocarbon receptor (AhR) is essential in immune homeostasis via modulation of pro‐inflammatory cytokines production and indoleamine 2,3‐dioxygenase (IDO). In this study, it is demonstrated that *P. gingivalis* may regulate AhR signalling in periodontitis, which provides a potential target for further immune regulation studies in periodontitis. Experimental periodontitis was induced in C57BL/6 mice by silk ligature and *P. gingivalis* oral inoculation. The alveolar bone resorption was examined using Micro‐CT. Histological structures were observed and related cytokines involved in AhR signalling pathway were analysed. RAW264.7 cells were pretreated with AhR agonist (FICZ) and antagonist (CH223191) and infected with *P. gingivalis* subsequently. The levels of IDO, AhR and other related cytokines were measured. To demonstrate IDO activity, the concentrations of tryptophan (Trp) and kynurenine (Kyn) were assessed by HPLC. Histological analysis of periodontitis mice showed distinct alveolar bone resorption and inflammatory cell infiltration. The level of AhR and its downstream target factors were significantly decreased in inflamed gingival tissue. Furthermore, RAW 264.7 cells incubated by *P. gingivalis* exhibited increased pro‐inflammatory cytokines production and decreased AhR, CYP1A1, CYP1B1, and IDO expression. Decreased IDO activity was observed as decreased Kyn/Trp ratio in the supernatant. Moreover, FICZ decreased the pro‐inflammatory cytokines levels in *P. gingivalis* infected cells. It is concluded that *P. gingivalis* may promote inflammatory responses via inhibiting the AhR signalling pathway in periodontitis.

## Graphical abstract

The schematic figure illustrating *P. gingivalis* inhibits Aryl hydrocarbon receptor (AhR) signalling pathway in periodontitis. *P. gingivalis* infection suppressed AhR and its downstream indoleamine 2,3‐dioxygenase (IDO) expression in periodontitis, which is responsible for the degradation of tryptophan (Trp) to kynurenine (Kyn). The downregulation of AhR signalling may increase IL‐6 and IL‐1β production by activating NF‐κB and NLRP3 inflammasome.

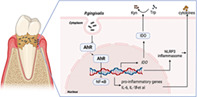

## 1 INTRODUCTION

Chronic periodontitis is a common inflammatory disease mainly because of opportunistic bacterial infections and leading to the destruction of periodontal tissues.^1^ Many studies have reported periodontitis may be associated with various systemic ailments, such as diabetes mellitus, atherosclerosis and so on.^2^, ^3^, ^4^ *Porphyromonas gingivalis* (*P. gingivalis*) is reported to be the main pathogen associated with chronic periodontitis, can activate inflammatory and immune responses in affected human periodontium.^5^

Aryl hydrocarbon receptor (AhR) is a transcription factor controlling the expression of many inflammatory factors. AhR signalling pathway is significantly suppressed in periodontitis.^6^ It is shown that AhR mainly located in the cytoplasm and translocate into nuclear when activated. It may block the binding sites of the nuclear factor kappa b (NF‐κB) and interfering its transcription activity, which finally inhibits pro‐inflammatory cytokine expression.^7^ Moreover, some researches showed that activated AhR signalling alleviated inflammation by suppressing NOD‐like receptor thermal protein domain associated protein 3 (NLRP3) inflammasome and its target cytokine IL‐1β.^8^, ^9^ AhR is a transcriptional factor that forms a heterodimer with AhR nuclear translocator (ARNT) protein, binding to the promoter of target genes to regulate its target genes' expression.^10^ CYP1A1 and CYP1B1 are two main AhR target genes, which are activated by AhR and can represent the level of AhR activation.^11^ AhR activation and its downstream signalling have been established as essential modulators for inflammatory regulation.^12^

On the other hand, AhR is essential in immune homeostasis by modulating indoleamine 2,3‐dioxygenase (IDO). IDO is a key immunoregulatory enzyme, which is responsible for the degradation of tryptophan (Trp) to kynurenine (Kyn).^13^, ^14^ The main function of IDO involved maintenance of T cells differentiation, especially the balance of Th17 cells and regulatory T cells (Tregs) differentiation from naïve T cells. Dysfunction of IDO might lead to a variety of immune‐mediated inflammatory diseases.^15^, ^16^ It is also reported that IDO may mediate Trp degradation in dendritic cells (DCs), macrophages, epithelial cells and various other cell types.^17^ Furthermore, in human and murine DCs, AhR was needed for the induction of IDO expression.^18^, ^19^ Additionally, AhR ligands could increase IDO expression and activity in murine bone marrow‐derived macrophages.^20^ The AhR‐IDO pathway has been involved in many inflammatory diseases.^21^, ^22^ Our previous study reported that the IDO activity and the Th17/Treg imbalance were related to *P. gingivalis* in atherosclerotic patients.^15^ However, whether *P. gingivalis* infection affects inflammation and immune imbalance via regulating the AhR signalling in periodontitis remains unclear and demands further investigation.

To figure out the role of *P. gingivalis* on AhR signalling pathway in periodontitis, we analysed the related inflammatory factors in *P. gingivalis* inoculated experimental periodontitis mice and investigate the potential mechanism using RAW 264.7 cells.

## 2 MATERIALS AND METHODS

### 2.1 Ethical statement

The protocols and experiments were approved by the Animal Ethics and Welfare Committee of Nanjing University (IACUC‐D2103024).

### 2.2 Experimental periodontitis mouse models

Twelve male C57BL/6 mice were used in this study. These mice were purchased from the Model Animal Research Center of Nanjing University. The mice were fed on standard diet and maintained under SPF conditions. They were allocated into two groups as the control group (*n* = 6, without ligature or oral inoculation of *P. gingivalis*) and the periodontitis group (*n* = 6, ligature and oral inoculation of *P. gingivalis*) randomly. The mouse model of periodontitis was established according to previous reference with minor modifications.^23^ Briefly, 8 weeks old mice were anaesthetized by injection with 4% chloral hydrate into the abdominal cavity after a week of acclimatizing. *P. gingivalis*‐soaked silk ligature was ligated around the left maxillary second molar for 2 weeks in periodontitis groups. In order to determine the role of *P. gingivalis*, 10^8^ CFU *P. gingivalis* in 0.1 ml 2% carboxymethylcellulose with phosphate‐buffered saline (PBS) or vehicle control were inoculated in the buccal surface of the mouse maxilla. The frequency of the inoculation was three times a week for 4 weeks. Mice were euthanized after the last management. Samples from maxilla and gingiva were obtained for further study.

### 2.3 Cell culture

RAW 264.7 cells, abelson murine leukaemia virus‐induced/immortalized male BALB/c mouse monocyte/macrophage cell line was purchased from American Type Culture Collection (ATCC). DMEM medium (Gibco) supplemented with 10% FBS were the main condition to culture cells. 6‐formylindolo[3,2‐b] carbazole (FICZ) (Sigma–Aldrich) or 2‐methyl‐2H‐pyrazole‐3‐carboxylic acid (2‐methyl‐4‐o‐tolylazo‐phenyl)‐amide (CH‐223191) (Selleck Chemicals LLC) were used to pretreat with RAW 264.7 cells for 12 and 24 h, respectively.

### 2.4 Bacterium culture

*P. gingivalis* ATCC 33277 was cultured in an anaerobic jar with an AnaeroPack (Thermo Fisher Scientific Inc.) generated anaerobic environment. Brain heart infusion (BHI) medium supplied with hemin and vitamin K was used to culture *P. gingivalis*. Three‐day‐old *P. gingivalis* at logarithmic growth phase were harvested by centrifugation at 5000 × *g* for 10 min. After resuspending with PBS, *P. gingivalis* was diluted with PBS to adjust the optical density to 0.8 at 660 nm, equalling to 10^9^ CFU/ml. *P. gingivalis* was incubated with cells at different multiplicities of infection (MOI).

### 2.5 Micro‐CT evaluation

The mice maxillae were dissected and fixed in 4% paraformaldehyde. Then, a micro‐CT scanning system (Bruker, Billerica, MA) was used to evaluate alveolar bone resorption. Parameters were as follows: 360° rotation angle, 50 kV X‐ray tube voltage, 445 μA tube current, 265 ms exposure time, 18 μm scanning layer thickness. All images were reconstructed using NRecon software and 3D models were obtained by CTVox and CTAn software. To assess the level of alveolar bone resorption, the length between cementum‐enamel junction (CEJ) and alveolar bone crest (ABC) was measured at the mesial and distal site of the left maxillary second molar.

### 2.6 Histology staining

The maxilla specimens were decalcified in 10% EDTA. Four weeks later, the specimens were dehydrated in progressively increased concentration ethanol and embedded in paraffin blocks. Sections were obtained every 4 μm cut along the long axis of molars. Next, HE staining was performed with haematoxylin and eosin.

### 2.7 Immunohistochemical (IHC) analysis

The paraffin tissues of the gingival tissues were hydrated and blocked with serum. Sections were incubated with anti‐IL‐6, anti‐IL‐1β and anti‐TNF‐α (Servicebio) antibodies respectively. After washing in PBS, HRP‐conjugated secondary antibody was incubated with sections, followed by 3,3′‐diaminobenzidine staining. Finally, counterstaining was performed using haematoxylin.

### 2.8 Quantitative real‐time PCR (qRT‐PCR)

The maxillae and gingival tissues were dissected carefully using microsurgical tweezers. RAW 264.7 cells were collected. RNA was extracted using RNA isolater Total RNA Extraction Reagent (Vazyme). After reverse transcription, cDNA was performed by qRT–PCR with an ABI Step‐one Plus system (Thermo Fisher Scientific Inc.). All gene expressions were calculated using $2^{-△△\mathrm{CT}}$ method. All primers sequences in this study were synthesized in GenScript Biotech and listed in Table 1.

**TABLE 1:** Primer sequences for genes targeted in qRT‐PCR

Gene	Forward primer (5′–3′)	Reverse primers (5′–3′)
*AHR*	AGCCGGTGCAGAAAACAGTAA	AGGCGGTCTAACTCTGTGTTC
*CYP1A1*	GACCCTTACAAGTATTTGGTCGT	GGTATCCAGAGCCAGTAACCT
*CYP1B1*	CACCAGCCTTAGTGCAGACAG	GAGGACCACGGTTTCCGTTG
*IDO1*	GCTTTGCTCTACCACATCCAC	CAGGCGCTGTAACCTGTGT
*IL1B*	GTCGCTCAGGGTCACAAGAA	TCAAAGCAATGTGCTGGTGC
*IL6*	CTGCAAGAGACTTCCATCCAG	AGTGGTATAGACAGGTCTGTTGG
*TNF*	GACGTGGAACTGGCAGAAGAG	TTGGTGGTTTGTGAGTGTGAG
*GAPDH*	TCTCCCTCACAATTTCCATCCCAG	GGGTGCAGCGAACTTTATTGATGG

### 2.9 Immunofluorescence (IF) analysis

The animal tissues were prepared as described above including fixation, decalcification, tissue embedding, and section. After deparaffinization and rehydration, samples were blocked with serum. RAW 264.7 cells were fixed in 4% paraformaldehyde. After permeabilization and blocking in 5% nonfat dry milk in PBS, the tissue sections or cultured cells were incubated with anti‐IDO antibody (Cell Signaling Technology) or anti‐AhR antibody (Proteintech). Next, tissue sections or cells were incubated with anti‐IgG antibody conjugated with Alexa Fluor 488 (Invitrogen). Rhodamine‐phalloidin (KeyGen) was used for stain actin filaments. 4′,6‐diamidino‐2‐phenylindole (DAPI) (Beyotime) was used to stain the nuclei. Pictures of immunofluorescent staining were visualized with confocal laser microscopy (LSM980, Zeiss).

### 2.10 Western blotting (WB)

To obtain protein from cells, RAW 264.7 cells were lysed by RIPA cell lysis buffer. SDS sample buffer was used to denature protein and then protein supernatants were heated at 95°C for 10 min. After quantification by the BCA method (Sigma–Aldrich), the proteins were separated by 10% precast gel (GenScript or KeyGen) via SDS–PAGE. The membranes were incubated with blocking buffer (NCM Biotech) after protein were transferred to PVDF membranes. Rabbit anti‐mouse AhR (Proteintech; or ImmunoWay Biotechnology) and rabbit anti‐mouse GAPDH (Cell Signaling Technology) were used. Images were visualized by the Tanon Gel Imaging System (Shanghai Tanon Co. Ltd.) or the Chemidoc MP Imaging System (Bio‐Rad).

### 2.11 High‐performance liquid chromatography (HPLC)

IDO activity in RAW 264.7 cells culture supernatants was determined via Trp and Kyn concentrations measurement by HPLC. The culture supernatant collected from *P. gingivalis* incubated RAW 264.7 cells was treated with 40% perchloric acid (Sinopharm Group) for protein precipitation. HPLC analysis was performed by an Agilent 1200 liquid chromatograph (Agilent) with a 5 μm SHISEIDO C18 (Shiseido) column. A solution of (A) pH 4.0, 15 mM sodium acetate buffer, and (B) 5% acetonitrile (95:5, v/v, Sigma–Aldrich) were applied at a flow rate of 0.8 ml/min as the mobile phase. All data were measured at 280 nm and 360 nm for Trp and Kyn, respectively.

### 2.12 ELISA

The concentration of cytokines IL‐6, IL‐1β and TNF‐α were determined by ELISA using respective ELISA kits (Neobioscience). *P. gingivalis* promotes pro‐IL‐1β synthesis instead of mature IL‐1β production unless the P2X7 receptor is activated by extracellular ATP (eATP).^24^ Therefore, to determine IL‐1β levels, ATP was used at 5 mM for an additional 50 min after *P. gingivalis* or AhR agonist and inhibitor to activate inflammasome complex.

### 2.13 Statistics

All in vitro experiments were repeated in three times independently. Results were shown as mean ± SD. A Shapiro–Wilk test was applied to decide the normality of data. Analysis of variance with Bonferroni correction or non‐parametric Wilcoxon test was operated under normal and non‐normal distributions. Student's *t*‐test or One‐way ANOVA with Bonferroni correction was performed to analysis the statistical significance among two or multiple groups. *p* value <0.05 was indicated statistically significant. SPSS 17.0 software was used to perform data analysis.

## 3 RESULT

### 3.1 *P. gingivalis* may suppress the AhR signalling pathway in mice periodontitis model

**FIGURE 1:**
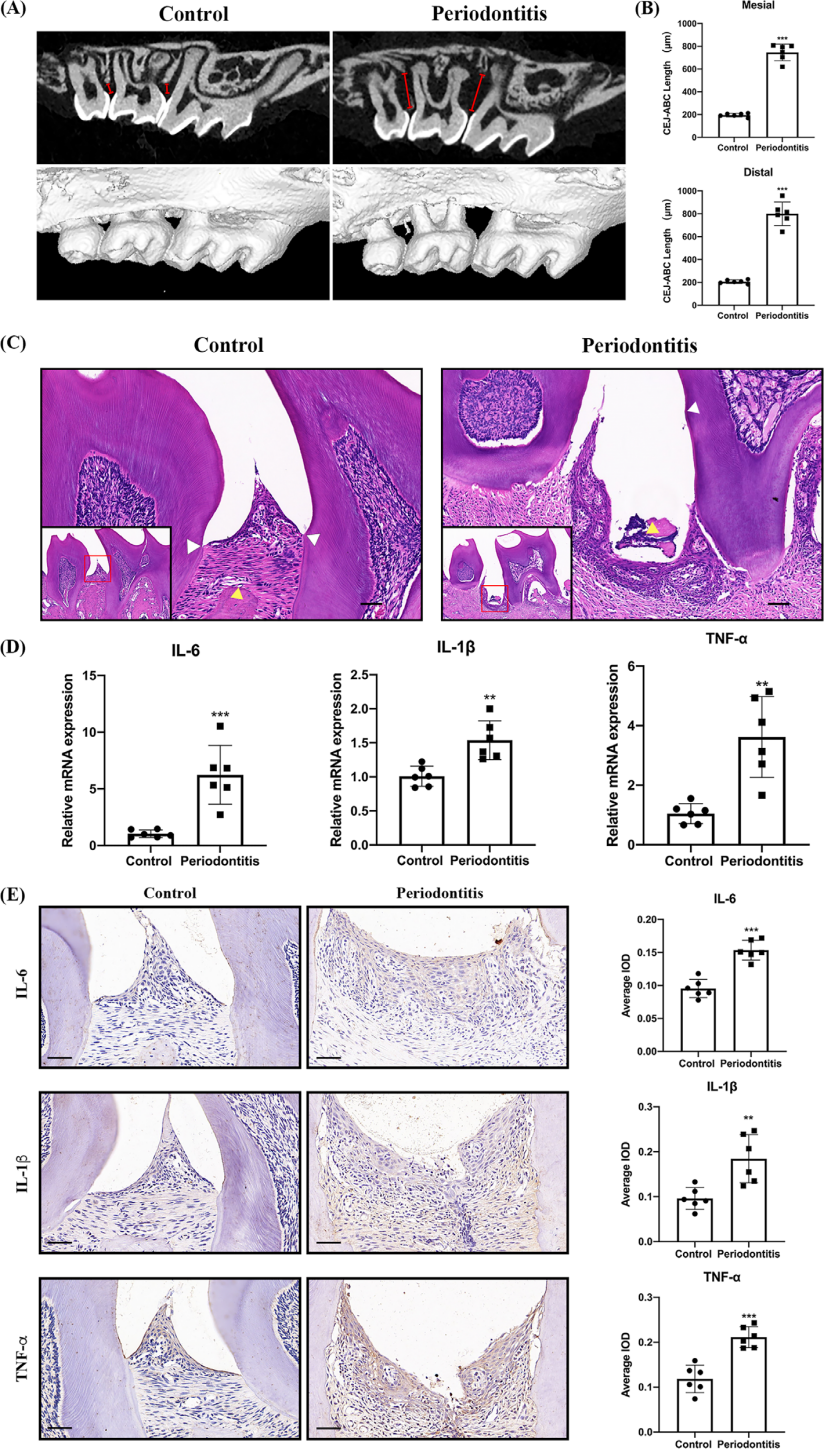
Alveolar bone resorption, inflammatory cells infiltration and inflammatory factor levels in periodontitis mouse model. Eight‐week‐old C57BL/6 mice were challenged by silk thread ligation with *Porphyromonas gingivalis* daubing in the left maxillary second molar for 4 weeks. (A) Periodontal bone was observed by the Micro‐CT. (B) Alveolar bone loss was represented by the distance of CEJ‐ABC. (C) Representative picture of H&E staining in periodontal tissues. (D, E) The gene and protein expression of IL‐6, IL‐1β and TNF‐α in gingival tissue were examined by qRT‐PCR and IHC. White arrows, CEJ; yellow arrows, ABC. *p < 0.05, **p < 0.01, ***p < 0.001

**FIGURE 2:**
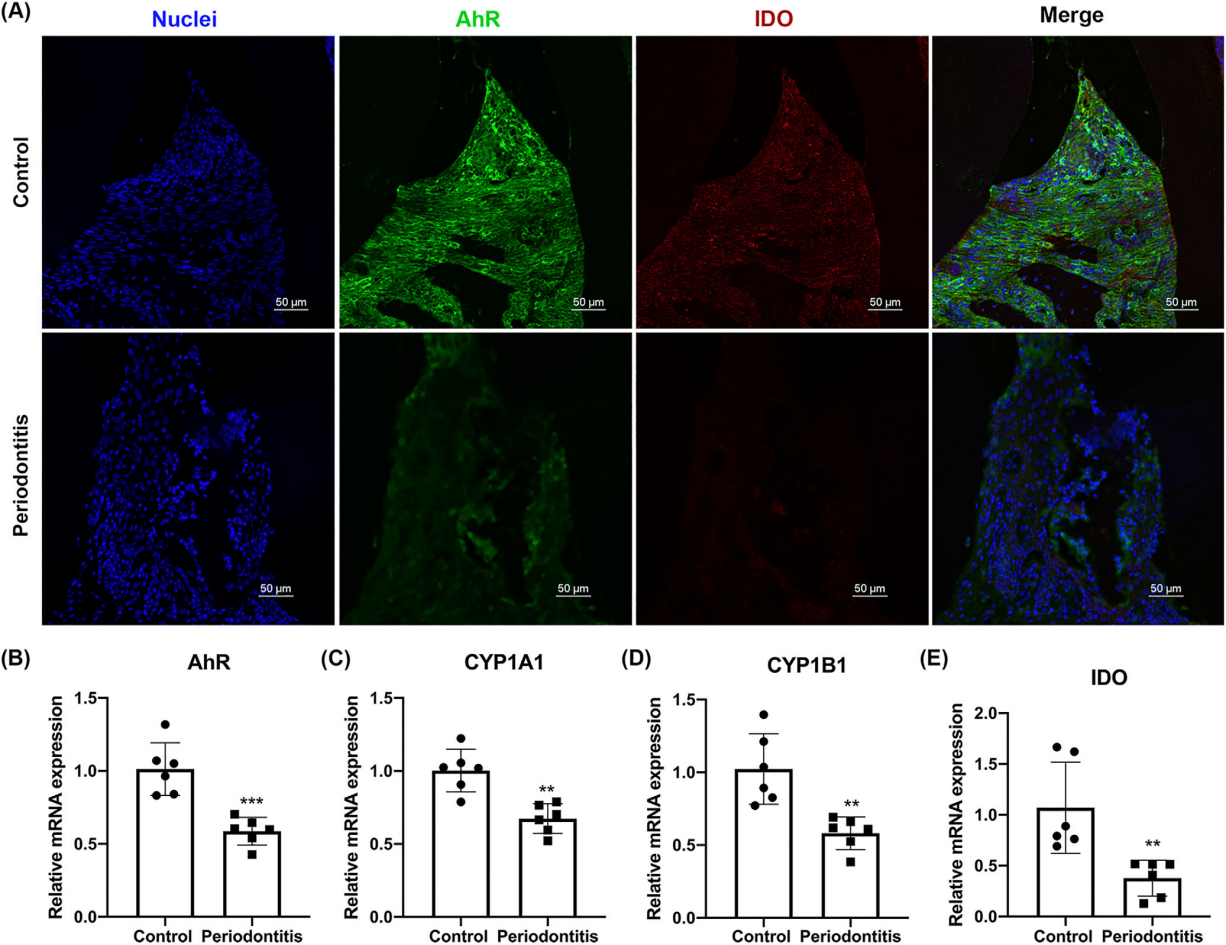
The expression of the AhR signalling pathway in gingival tissues. (A) Immunofluorescence analysis of AhR and IDO expression in gingival tissues. AhR are shown in green. IDO are stained in red. Nuclei are shown in blue stained with DAPI. (B–E) mRNA expression levels of AhR, CYP1A1, CYP1B1 and IDO from gingival tissues measured by qRT–PCR. *p < 0.05, **p < 0.01, ***p < 0.001

To clarify the involvement of the AhR signalling pathway in the progression of periodontitis, mice periodontitis model was established using C57BL/6 mice. Micro‐CT results showed that the mouse periodontitis model induced by ligature and *P. gingivalis* infection resulted in a significant alveolar bone loss (Figure 1A,B). H&E staining showed there were more inflammatory cellular infiltration in periodontitis (Figure 1C). The expression of IL‐6, IL‐1β, and TNF‐α determined by qRT‐PCR and IHC were greatly increased in the inflamed gingiva (Figure 1D,E). On the other hand, the immunofluorescent analysis represented that the expression of AhR and IDO were decreased in inflamed gingival tissues compared with control group (Figure 2A). Similarly, gene expression of AhR, CYP1A1, CYP1B1, and IDO analysed by qRT‐PCR were also decreased in periodontitis mice (Figure 2B–E).

### 3.2 *P. gingivalis* stimulates the production of pro‐inflammatory cytokines in murine monocyte–macrophage cell line

**FIGURE 3:**
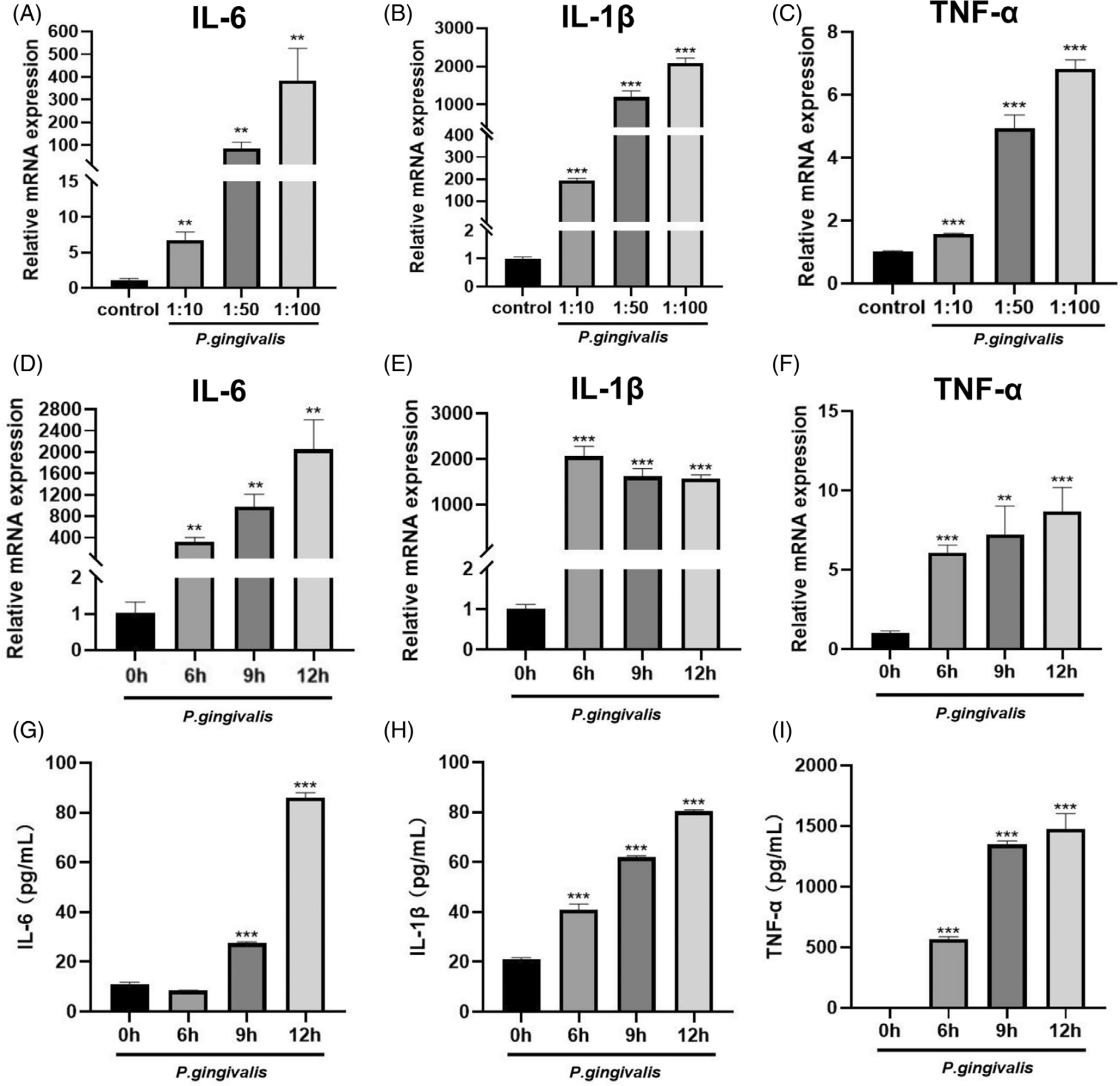
*Porphyromonas gingivalis* stimulated the expression of proinflammatory cytokines IL‐6, IL‐1β and TNF‐α in RAW 264.7 cells. (A–C) RAW 264.7 cells were infected with *P. gingivalis* at MOI of 10, 50 or 100 for 6 h, the gene expression of pro‐inflammatory factors IL‐6, IL‐1β and TNF‐α were measured by qRT‐PCR. (D–F) RAW 264.7 cells were incubated with *P. gingivalis* at MOI of 100 for 0, 6, 9 or 12 h, the gene expression of IL‐6, IL‐1β and TNF‐α were measured by qRT‐PCR. (G–I) The concentration of IL‐6, IL‐1β and TNF‐α in the culture supernatant of RAW 264.7 cells infected with *P. gingivalis* at MOI of 100 over 0, 6, 9 or 12 h. **p < 0.01, ***p < 0.001 vs. control

To determine the impact of *P. gingivalis* on inflammatory responses, a murine monocyte–macrophage cell line, RAW 264.7 cells were applied in the study. Cells were incubated with *P. gingivalis* at different MOI of 10, 50 and 100 for different time (6, 9 and 12 h) and the expression of IL‐6, IL‐1β and TNF‐α were determined. According to the results, the production of these cytokines was increased with higher *P. gingivalis* MOI and longer time incubation. Furthermore, correspondingly enhanced cytokines production was detected by ELISA in the *P. gingivalis* treated RAW 264.7 cells culture supernatants (Figure 3).

### 3.3 AhR may regulate IDO expression in RAW264.7 cells

**FIGURE 4:**
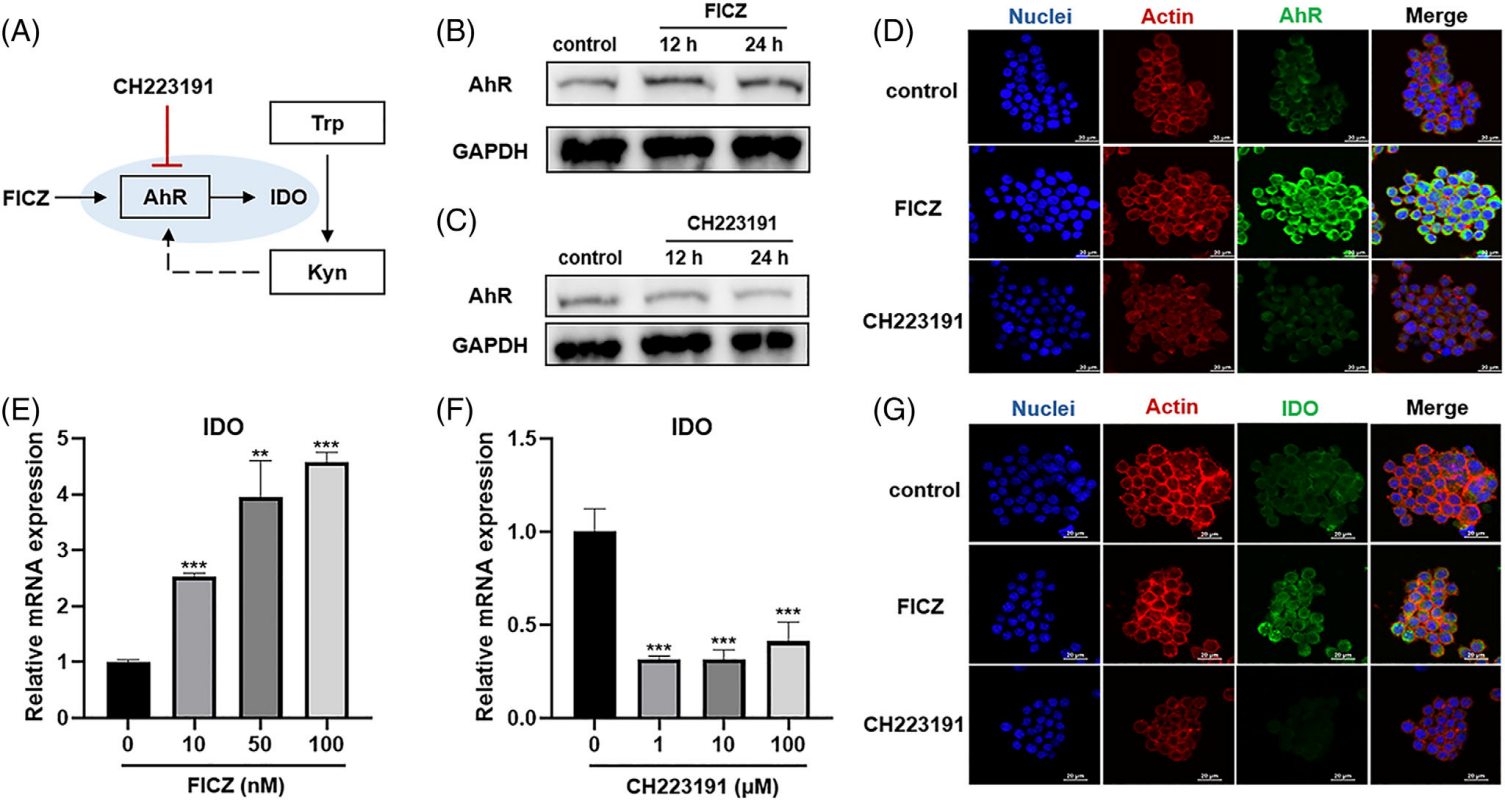
AhR regulates the expression of IDO in RAW 264.7 cells. (A) Schematic of the AhR/IDO signalling pathway. (B, C) RAW 264.7 cells were treated with 0.1 μM FICZ or 10 μM CH‐223191 for 12 h or 24 h, with AhR levels were examined by western immunoblotting. (D, G) Confocal laser scanning microscopy images showing localization of AhR and IDO in RAW 264.7 cells treated by FICZ or CH‐223191 for 24 h (DAPI: DNA; fluorescein isothiocyanate: AhR or IDO; rhodamine‐phalloidin: Actin). (E, F) Relative mRNA expression levels of IDO from RAW 264.7 cells treated by FICZ (10, 50, 100 nM) or CH‐223191 (1, 10, 100 μM) for 24 h. **p < 0.01, ***p < 0.001 vs. control

The detailed mechanism of AhR‐IDO signalling remained unclear in RAW 264.7 cells. To investigate the role of AhR in regulating IDO, the cells were incubated with 0.1 μM FICZ (AhR agonist) or 10 μM CH‐223191 (AhR antagonist) for 12 and 24 h, respectively. Expression of AhR was examined by WB and IF analysis (Figure 4B–D). The expression of AhR was increased in cytosol in FICZ incubated cells. The protein level of AhR was slightly increased with FICZ treatment and decreased with CH‐223191 treatment. Furthermore, IDO expression detected by qRT‐PCR and immunofluorescence assay showed IDO was highly elevated in FICZ‐treated group but greatly reduced in the CH‐223191 treated group (Figure 4E,F). Notably, immunofluorescence analysis confirmed the cytoplasmic localization of IDO and the corresponding IDO fluorescence intensity match with the FICZ/CH‐223191 induced changes observed under qRT–PCR (Figure 4G). These findings demonstrated that AhR exerted a positive regulatory effect on IDO expression in RAW 264.7 cells.

### 3.4 *P. gingivalis* reduces the expression, production and hence tryptophan degradation of IDO in RAW 264.7 cells

**FIGURE 5:**
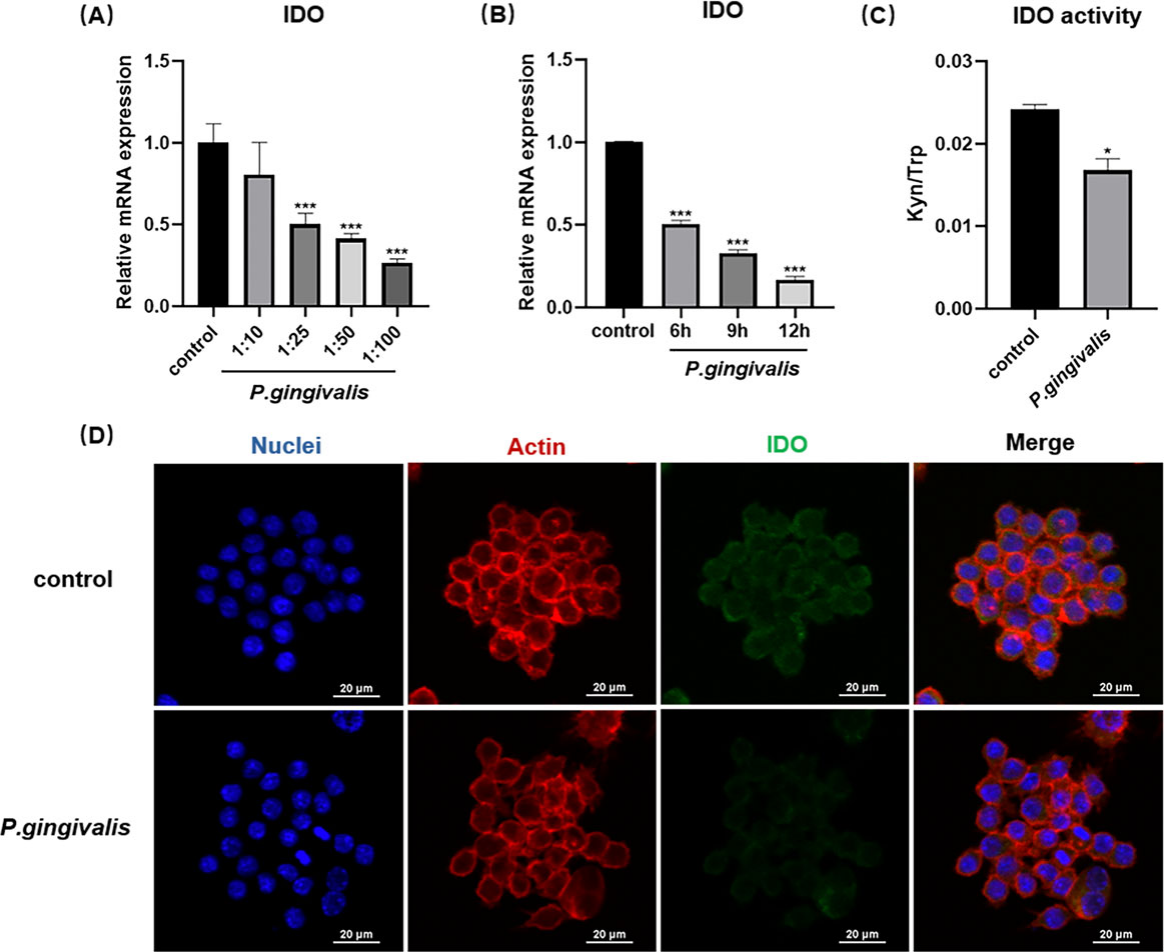
*Porphyromonas gingivalis* reduces the expression and activity of IDO from RAW 264.7 cells. (A) IDO mRNA expression from RAW 264.7 cells examined by qRT–PCR after *P. gingivalis* challenged at MOI of 10, 25, 50 or 100 for 12 h. (B) IDO gene expression from RAW 264.7 cells determined by qRT–PCR after *P. gingivalis* challenged at 100 MOI for 6, 9 or 12 h. (C) High‐performance liquid chromatography detected kynurenine (Kyn) and tryptophan (Trp) concentrations depicted as Kyn/Trp in RAW 264.7 cells culture supernatant after *P. gingivalis* infection at 100 MOI for 24 h. (D) Confocal laser scanning microscopy images showing localization of IDO (DAPI: DNA; fluorescein isothiocyanate: IDO; rhodamine‐phalloidin: Actin). *p < 0.05, ***p < 0.001 vs. control

To discuss the effect of *P. gingivalis* on IDO production and function, cells were incubated with *P. gingivalis* at MOI of 10, 25, 50, 100 for 12 h, or MOI of 100 for 6, 9 and 12 h. Results showed IDO was reduced with higher MOI and time (Figure 5A,B). We further measured IDO enzymatic activity and protein expression after bacterial infection with an optimal MOI of 100. The Kyn/Trp ratio reduced at 24 h after infection (Figure 5C). Moreover, immunofluorescence analysis showed that IDO fluorescence intensity was decreased in *P. gingivalis* treated cells (Figure 5D). These results suggested *P. gingivalis* infection could downregulate the IDO expression and therefore impacted upon the production and biological activities of IDO.

### 3.5 *P. gingivalis* may regulate IDO levels by influencing AhR activity

**FIGURE 6:**
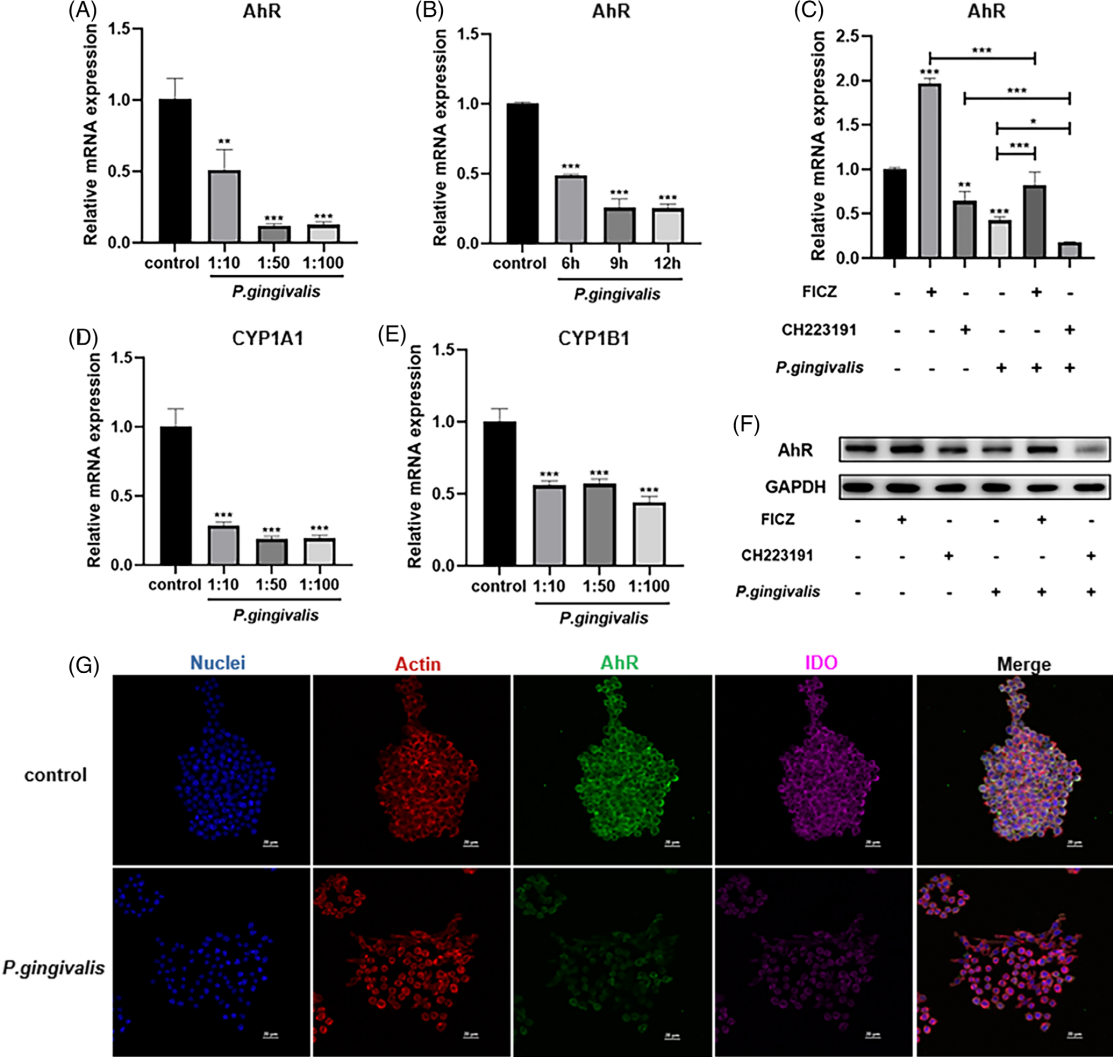
*Porphyromonas gingivalis* regulates IDO levels in RAW 264.7 cells putatively via influencing the AhR activity. (A) AhR mRNA expression were detected in RAW 264.7 cells after *P. gingivalis* infection at MOI of 10, 50 or 100 for 12 h. (B) AhR mRNA expression were detected in RAW 264.7 cells infected with *P. gingivalis* at MOI of 100 for 6, 9 or 12 h. (C) Relative mRNA expression levels of AhR from RAW 264.7 cells were treated with 0.1 μM FICZ or 10 μM CH‐223191 for 24 h with or without *P. gingivalis* at 100 MOI for 12 h. (D, E) RAW 264.7 cells' *CYP1AI* or *CYP1BI* mRNA expression were measured by qRT–PCR to represent target genes for AhR transcriptional activation after *P. gingivalis* challenged at 10, 50 or 100 MOI for 12 h. (F) AhR protein expression was measured by WB upon same *P. gingivalis* challenge described in panel C. (G) Confocal laser scanning microscopy images showing co‐localization of AhR and IDO (DAPI: DNA; fluorescein isothiocyanate: AhR; Cy‐Chrome 5: IDO; rhodamine‐phalloidin: Actin). *p < 0.05, **p < 0.01, ***p < 0.001 vs. control or indicated otherwise

Having established that AhR indeed contributes to regulating of IDO expression and production, we next investigated whether the negative outcomes of IDO upon *P. gingivalis* infection could possibly be also related to AhR regulation. AhR levels after *P. gingivalis* infection at MOI of 10, 50 100 for 12 h, or MOI of 100 for 6, 9 and 12 h in RAW 264.7 cells were measured respectively. It was shown that AhR gene expression could be suppressed by *P. gingivalis* infection (Figure 6A,B). Furthermore, we observed decreases in the expressions of the AhR transcriptional targets CYP1A1 and CYP1B1 in *P. gingivalis*‐infected cells (Figure 6D,E). To further determine the effects of *P. gingivalis* challenge on AhR production in the process, cells were firstly stimulated with AhR agonist 0.1 μM FICZ or antagonist 10 μM CH‐223191 for 24 h and subsequently infected with *P. gingivalis* for 12 h at MOI of 100. Results revealed that gene and protein expressions of AhR could be suppressed by *P. gingivalis* infection while AhR was activated or inhibited by FICZ or CH‐223191 (Figure 6C,F). Immunofluorescent analysis demonstrated that under *P. gingivalis* infection, cytosol expressions of both AhR and IDO proteins were greatly reduced (Figure 6G), indicating *P. gingivalis* may suppress IDO production could be potentially mediated through AhR inhibition.

### 3.6 AhR agonist downregulates inflammatory cytokines production in RAW 264.7 cells upon *P. gingivalis* challenge

**FIGURE 7:**
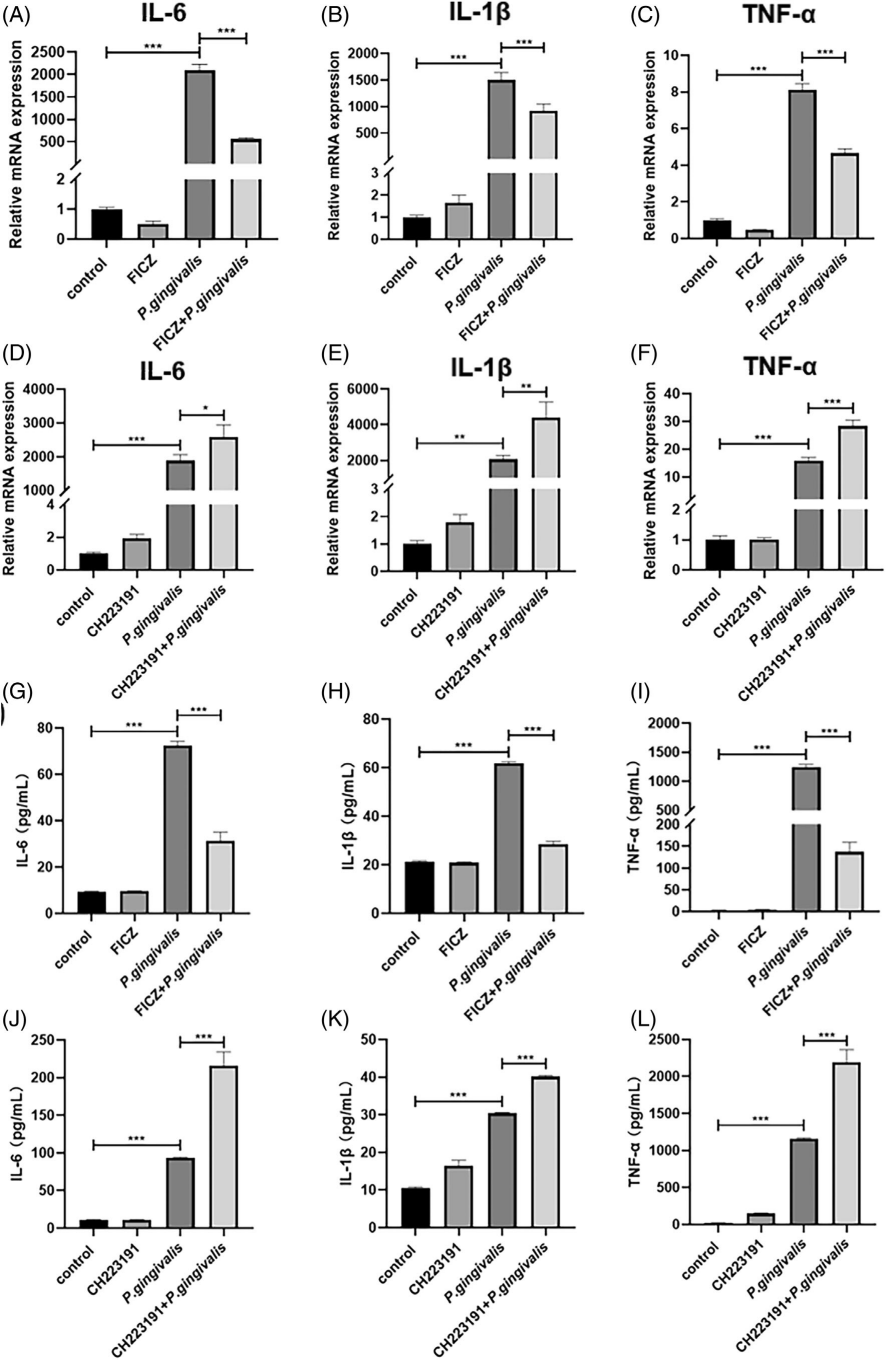
AhR agonist downregulates inflammatory responses induced by *Porphyromonas gingivalis* in RAW 264.7 cells. RAW 264.7 cells treated with 0.1 μM FICZ or 10 μM CH‐223191 for 24 h, following with or without *P. gingivalis* at MOI of 100, 12 h. RAW 264.7 cells mRNA expression, measured by qRT–PCR, concerning IL‐6 (A, D), IL‐1β (B, E), or TNF‐α (C, F) as well as corresponding cytokines in culture supernatant assayed by ELISA (G–L). *p < 0.05, **p < 0.01, ***p < 0.001

To further investigate whether *P. gingivalis*‐induced inflammatory cytokines were related to the AhR, RAW 264.7 cells were incubated with 0.1 μM FICZ or 10 μM CH‐223191 for 24 h and subsequently stimulated with *P. gingivalis* for 12 h at MOI of 100. Data showed that *P. gingivalis*‐induced IL‐6, IL‐1β and TNF‐α gene expression and protein in culture supernatant production were affected by FICZ pre‐incubation, while the same was clearly enhanced by CH‐223191 pretreatment significantly (Figure 7). Thus, it is indicated the *P. gingivalis‐*induced inflammatory cytokines production could potentially play roles in AhR‐related pathways.

## 4 DISCUSSION

*P. gingivalis* is an anaerobic bacteria and a key periodontal pathogen, which could produce a series of virulent factors such as gingipains, fimbriae, and lipopolysaccharide (LPS). *P. gingivalis* infection could interfere the host immune homeostasis and cause local immune response dysregulation in the infected periodontium.^25^, ^26^ In the present study, AhR and its direct targets CYP1A1 and CYP1B1 were reduced in *P. gingivalis‐*infected experimental periodontitis mice model. It is consistent with study reported by Huang Jin et al that suppressed AhR signalling was found in periodontitis mice.^6^ In order to elucidate the mechanism of *P. gingivalis* on AhR in the pathogenesis of periodontitis, the effect of *P. gingivalis* on AhR signalling was determined in RAW 264.7 cells. It is a monocyte/macrophage, which plays a key role in regulating immune response. It is also one of the first types of immune cells to react to microorganisms in periodontitis. Macrophages can not only release a variety of inflammatory mediators, but also present antigen to T cells to activate the immune response.^27^, ^28^ In our study, *P. gingivalis* could greatly downregulate the expression of AhR, CYP1A1, and CYP1B1. Moreover, IL‐6, IL‐1β, and TNF‐α expression were increased significantly in *P. gingivalis*‐infected RAW264.7 cells.

The effect of AhR agonist FICZ and antagonist CH‐233191 on CYP1A1 and CYP1B1 was clear nowadays. However, the function of FICZ and CHI‐223191 on AhR expression has been reported in many cells and inflammatory animal models, but the effects were inconsistent in different cells. So far, the role of FICZ and CH‐223191 in RAW 264.7 cells was not clear. In our study, the gene expression and nuclear translocation of AhR were increased when incubated with FICZ. The total AhR protein level including in both nuclear and cytosol in RAW 264.7 cells was slightly increased. CH‐223191 decreased the expression of AhR. It is similar to some studies that the agonist FICZ promoted AhR nuclear translocation and the protein expression of AhR was increased. CH‐223191 downregulated AhR expression.^29^, ^30^ As observed in the current study, AhR agonist pre‐incubation reversed the inflammatory responses induced by *P. gingivalis* while AhR antagonist upregulated pro‐inflammatory cytokines expression indicating AhR signalling perhaps played part in the cytokine modulation/production.

Additionally, it has been shown that activated AhR signalling inhibits IL‐6 and IL‐1β production by inhibiting NF‐κB and NLRP3 inflammasome.^31^, ^32^ Both NF‐κB and NLRP3 inflammasome are pivotal regulators of inflammation in periodontitis.^33^ It has been well‐documented that NF‐κB up‐regulates the transcription of the NLRP3 inflammasome formation and the gene transcription of pro‐inflammatory factors by binding to the κB site located in the promoter of NLRP3. Recent studies have found that AhR exerts a repression on NF‐κB transcription activity. After AhR translocating into nuclear, ARNT binds to the XRE regions located at κB site, leading to the inhibition of NF‐κB transcription activity. Finally, NLRP3 inflammasome activation is inhibited and the production of subsequent proinflammatory cytokine is decreased. The results in our study showed *P. gingivalis*‐induced IL‐6, IL‐1β and TNF‐α gene expression and protein production were affected by AhR agonist. According to the present study and previous reports, we indicated that *P. gingivalis* may regulate inflammatory mediators related to the NF‐κB and NLRP3 pathway by inhibiting AhR signalling pathway. Hence AhR signalling would be an interesting and important direction for future exploration.

The expression and activity of IDO were significantly decreased in both periodontal tissue of periodontitis mice, and a monocyte–macrophage cell line infected with *P. gingivalis*. It is worth mentioning that AhR may be an important modulator of IDO enzymatic activity.^34^ Previous studies have shown that there is an autocrine IDO‐Kyn/AhR‐IDO positive feedback loop in dendritic cells and AhR could contribute to the expression of IDO through this mechanism.^34^, ^35^ In Pallotta's study, Kyn–bound AhR was shown to promote IDO phosphorylation by inducing IL‐6 and suppressor of cytokine signalling‐3 activity, trigging the ubiquitination and proteasomal degradation of IDO.^36^ The relationship between IDO and AhR remained rather complex. The current findings demonstrated that enhancing AhR activity upregulated IDO expression while suppressing AhR activity exerted the opposite effects. Overall, the concept of AhR promoting expression of IDO evolved, despite understanding regarding its mechanism of action, remained not fully elucidated. Furthermore, given the essential role of AhR in regulating IDO levels as well as the prior knowledge that AhR can modulate IDO enzymatic activity,^37^ the repression of IDO in RAW 264.7 cells caused by *P. gingivalis* infection appears to be in part mediated via AhR.

The enzyme IDO plays key roles in regulating immunity and inflammation through the degradation of Trp into Kyn and other downstream metabolites.^13^ It has been previously shown that Trp depletion via IDO‐activated Tregs and inhibited Th17 cells attenuated proinflammatory responses in various inflammatory diseases.^38^, ^39^ Several cell types exert anti‐inflammatory effects via IDO expression induction.^40^ Our results revealed that *P. gingivalis* markedly reduced the IDO, suggesting that the periodontal pathogen may exert its pathogenicity via the IDO‐related pathway. In fact, Palm et al. reported that *P. gingivalis* and its virulence factor gingipains suppressed the expression of IDO in human gingival fibroblasts, which resulted in a reduction in the host response that could further establish pathogenicity of *P. gingivalis* against host immunity.^41^ Such a finding was in line with the results of the current report that *P. gingivalis* suppressed the expression of IDO in RAW 264.7 cells. However, there is another study reported that *P. gingivalis* LPS can induce IDO expression.^42^ In our study, viable *P. gingivalis* was used with which the LPS would be readily exposed to the RAW 264.7 cells tested. Gingipains may be the main virulence factor involved in IDO regulation. The biological relevance of *P. gingivalis* LPS and gingipain upon RAW 264.7 biology remained to be determined. Based on the available literature, we reported the mechanism of how *P. gingivalis* could interfere with macrophagic cells' IDO biology via AhR. Considering the relevance of the AhR‐mediated inflammation regulation as well as the AhR‐IDO‐Kyn/Trp T cells modulation pathways, this report indicated plausible pathogenic mechanisms through which *P. gingivalis* disrupts host immune regulation. Considering the limited experimentation reported in the current report, further experiments are warranted to explore the complete picture concerning the influences of *P. gingivalis* upon adaptive cellular immune responses via AhR‐IDO‐Kyn/Trp T cell modulation.

**FIGURE 8:**
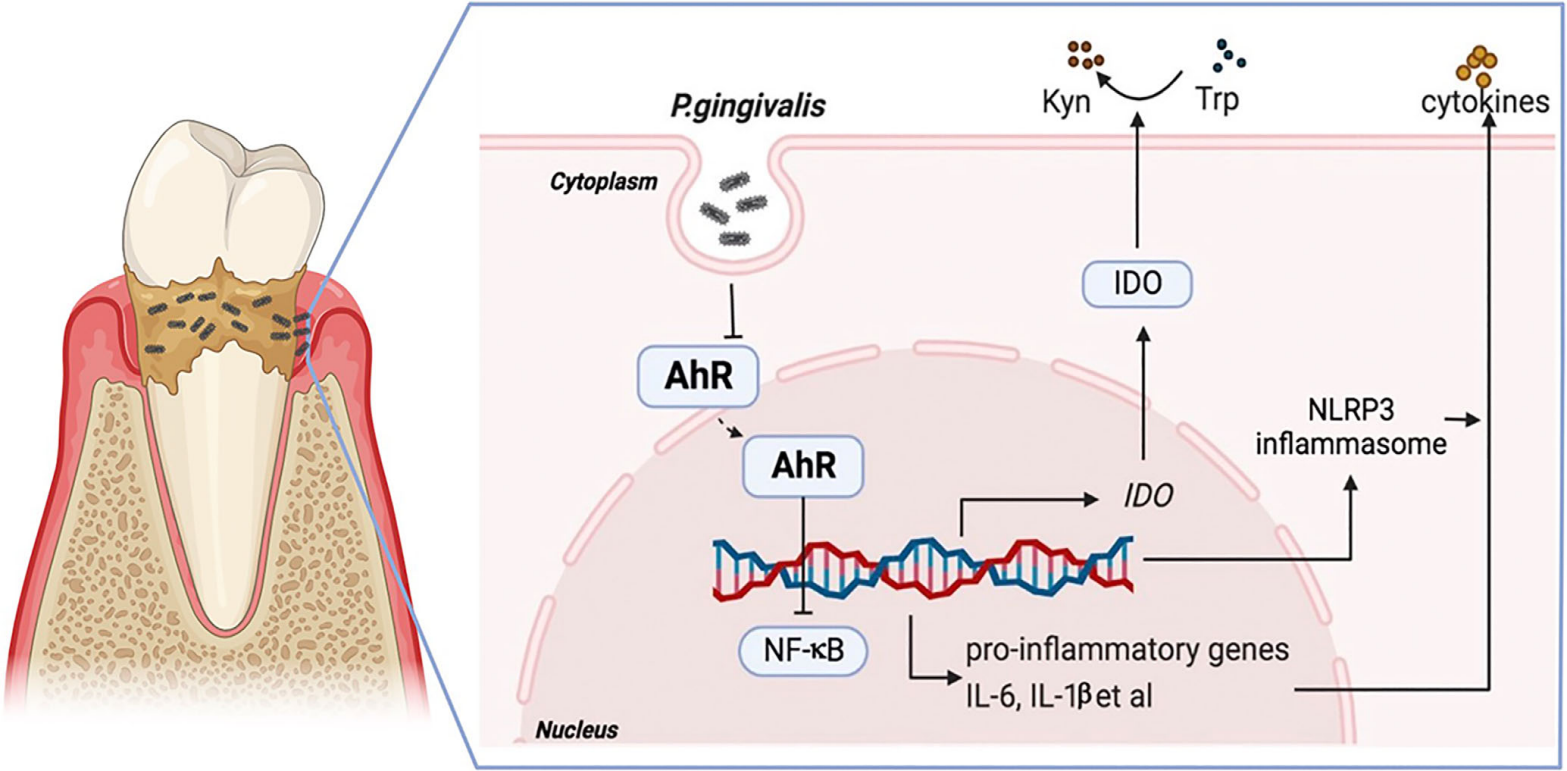
The schematic figure illustrating *Porphyromonas gingivalis* inhibit Aryl hydrocarbon receptor (AhR) signalling pathway in periodontitis. *P. gingivalis* infection suppressed AhR and its downstream indoleamine 2,3‐dioxygenase (IDO) expression in periodontitis, which is responsible for the degradation of tryptophan (Trp) to kynurenine (Kyn). The downregulation of AhR signalling may increase IL‐6 and IL‐1β production by activating NF‐κB and NLRP3 inflammasome.

The current report showed that *P. gingivalis* infection suppressed AhR and its downstream IDO expression in periodontitis, which is responsible for the degradation of Trp to Kyn. The downregulation of AhR signalling may increase the production of IL‐6, IL‐1β et al. cytokines by activating NF‐κB and NLRP3 activity (Figure 8). Overall, this study provides evidence supporting perhaps a novel mechanism by which *P. gingivalis* could induce inflammatory responses in periodontitis by suppressing the AhR signalling pathway.

## AUTHOR CONTRIBUTIONS

Jie Yang and Weibin Sun did the study design. Ting Hao and Rui Zhang conducted the study and collected the data. Ting Hao drafted the manuscript. Tian Zhao, Wai Keung Leung and Jie Yang revised manuscript content. Juan Wu, Wai Keung Leung and Jie Yang devoted to project administration. Jie Yang and Weibin Sun contributed to funding acquisition. Ting Hao, Jie Yang and Weibin Sun take responsibility for the integrity of the data analysis. All authors agreed to the published version of the manuscript.

## CONFLICT OF INTEREST

The authors declare no conflict of interest.

## Data Availability

The data that support the findings of this study are available from the corresponding author upon reasonable request.
